# Malignant melanoma bone marrow infiltration induced coagulation dysfunction and spinal epidural haematoma with paraplegia: a case report and literature review

**DOI:** 10.3389/fmed.2025.1601774

**Published:** 2025-10-27

**Authors:** Songhua Liu, Baode Zhang, Yan Shi, Jing Guo, Weihua Yin, Qinqin Xu, Jinzhu Liu, Shaoxiong Min

**Affiliations:** ^1^Shenzhen Key Laboratory of Spine Surgery, Department of Spine Surgery, Peking University Shenzhen Hospital, Shenzhen, China; ^2^Department of Spine Surgery, Shenzhen Xinhua Hospital, Shenzhen, China; ^3^Department of Pathology, Peking University Shenzhen Hospital, Shenzhen, China

**Keywords:** bone marrow infiltration, coagulation dysfunction, case report, malignant melanoma, paraplegia, spinal epidural haematoma

## Abstract

Malignant melanoma bone marrow infiltration induced coagulation dysfunction and spinal epidural haematoma with paraplegia is extremely rare. It typically presents as anemia, coagulation dysfunction or disseminated intravascular coagulation, immune thrombocytopenia. In severe cases, it can lead to spinal epidural hematoma, compressing the spinal cord and nerve roots, resulting in motor and sensory dysfunction, and even paraplegia. Due to the rarity and complexity of this condition, it is prone to misdiagnosis or delayed diagnosis, ultimately leading to a poor prognosis. This paper reports the case of a 14-year-old female who was urgently admitted to the emergency department with low back pain for 1 month, accompanied by systemic mucous membranes bleeding and ecchymosis for half a month, numbness and incomplete paralysis in both lower limbs for 3 days. Laboratory tests indicated pancytopenia and abnormal coagulation function. Magnetic resonance imaging: T11-L1 epidural irregular abnormal signals in the spinal canal, with a range of about 16 mm × 17 mm × 65 mm. Further physical examination revealed that a large, cauliflower-like black mole on the right scalp, which had been present since childhood and had recurrently ulcerated without healing. Bone marrow aspiration biopsy confirmed the diagnosis of malignant melanoma with bone marrow infiltration, leading to coagulation dysfunction and SEH with paraplegia. However, despite 2 months of aggressive symptomatic and supportive treatment, the child ultimately succumbed to malignant melanoma forever. This report shares our experience with the diagnosis and treatment of this case, highlighting the necessity of thoroughly reassess the medical history and conduct a detailed physical examination.

## Introduction

Malignant melanoma (MM) is a highly malignant melanocytic tumor, accounting for 1–3% of all malignant tumors (1). Its main primary site is the skin tissue, and it can be widely metastasised throughlymph and blood circulation, and common metastatic sites include the lung, brain, liver, bone marrow or intestines (2, 3). Patients with malignant melanoma bone marrow infiltration usually present with anemia, coagulopathy or disseminated intravascular coagulation (DIC) and immune thrombocytopenia (ITP) (4–6). When coagulation dysfunction occurs, it may lead to bleeding in multiple parts of the body. Among them, spinal epidural hematoma (SEH) is a rare complication, that can cause compression of the spinal cord and nerve roots, resulting in motor and sensory dysfunction, and even paraplegia (7, 8). As far as we know, cases of coagulopathy secondary to MM bone marrow metastasis resulting in SEH have rarely been reported in the literature. The pathogenesis of SEH may be associated with local vascular damage and systemic coagulopathy secondary to MM bone marrow infiltration, however, the precise pathological mechanisms remain incompletely understood. The etiology of SEH is predominantly attributed to trauma, surgery, anesthesia, and other invasive procedures. Here, we report a case of a rare MM bone marrow infiltration induced coagulation dysfunction and SEH, which eventually developed into paraplegia. By detailed analysis of the diagnosis and treatment process, we aim to further explore the diagnosis and treatment strategies of complications related to MM bone marrow infiltration and provide a reference for clinical practice.

## Case report

**Figure 1:**
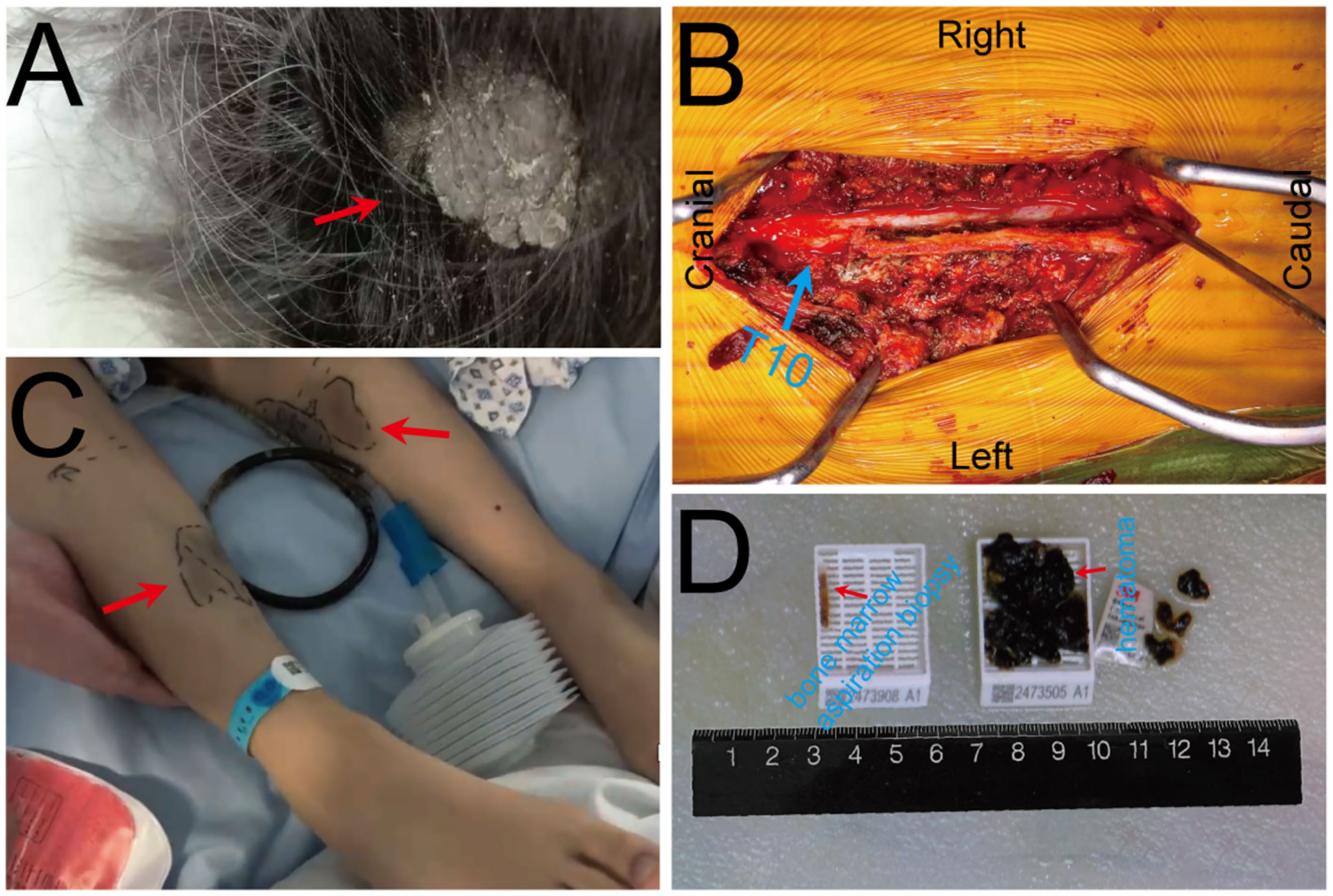
Pre-operative clinical and intra-operative images. **(A)** A large cauliflower-like black mole on the right scalp (arrow); **(B)** Intra-operative view of the surgical field with T10 vertebral body marked by blue. **(C)** Scattered bruising and ecchymosis on bilateral lower limbs (arrows); **(D)** Bone marrow aspiration biopsy specimen (left) and intra-operative evacuated hematoma specimen (right).

**Figure 2:**
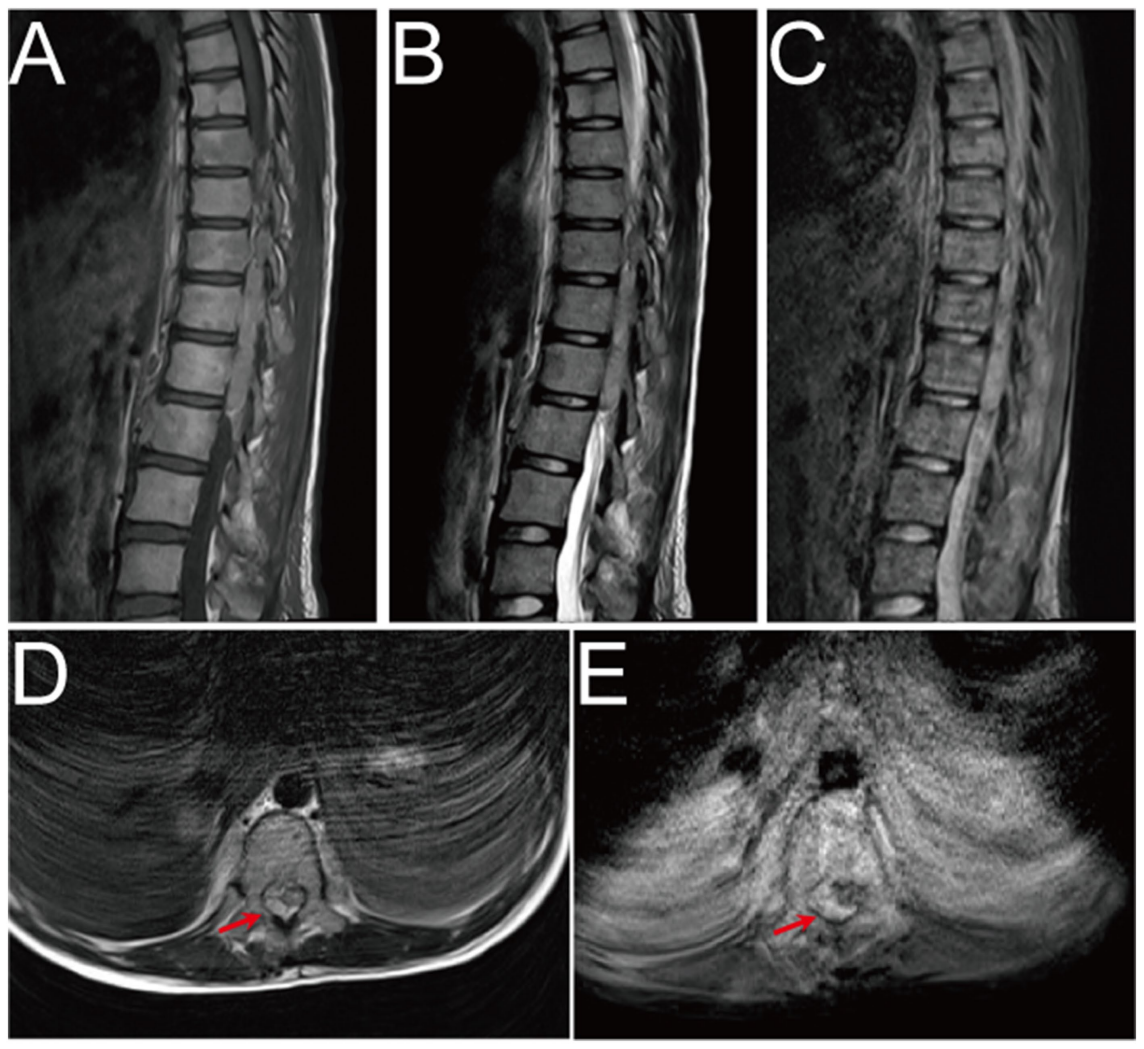
Pre-operative MRI image. **(A–E)** Pre-operative MRI showed patchy heterogeneous signals in the vertebral body and appendages, and epidural hematoma at the T10-L1 level. The hematoma was crescent-shaped, measuring approximately 16 mm × 17 mm × 65 mm. Both T1WI and T2WI showed hyperintensity, while fat-saturation proton density-weighted sequences showed hyperintensity.

On Oct. 23, 2024, a 14-year-old female was urgently admitted to the emergency department (ED) with low back pain for 1 month, accompanied by systemic mucous membranes bleeding and ecchymosis for half a month, numbness and incomplete paralysis in both lower limbs for 3 days. One month ago, the patient suffered from lumbar pain due to an accidental sprain while running. Half a month ago, the back pain was further worsened after acupuncture on the latissimus dorsi muscles on both sides of the spine, accompained by swelling of the lumbar and buttock muscles, ecchymosis, and active bleeding gums. Fever appeared 4 days ago, with a maximum temperature of 38.5 °C, accompanied by acute urinary retention, the back pain worsened, and progressive decline in muscle strength of both lower limbs. After symptomatic conservative treatment such as antibiotics, analgesics drugs, and blood product transfusion, there was failure improvement in symptoms. No other special treatment. Parents are healthy, and there is no family history of genetic disease or similar disease. Physical examination: Forced high fowler’s position (60°–90°), severe malnutrition, anemia, pale systemic skin and mucous membranes, scattered in large areas of bruising and ecchymosis, especially in the lower limbs, slightly convex skin surface (Figure 1C). Both frontal nasal cavities have been tamponade to stop bleeding, the lumbar and buttock muscles are slightly swollen, and subcutaneous hematoma has formed. The movement of thoracolumbar and both lower limbs was significantly limited, the thoracolumbar and back tenderness and percussion pain. The grade muscle strength of both upper limbs, iliopsoas muscle, quadriceps femoris, tibialis anterior and extensor hallucis longus were 5-/5, 3-/5, 3-/5, 0/5, and 0/5, respectively. Sensation in the perineum and below the plane of the inguinal region was significantly reduced. Active range of motion (AROM) in bilateral lower extremities: hip flexion 0°–45°, knee flexion 0°-90°, ankle dorsiflexion 0°. Brudzinski sign (−), Kernig sign (−), and Babinski sign (−), absent tendon reflexes, ankle and patellar clonus (The child was unable to tolerate physical examination due to severe pain.). Laboratory data: white blood cell (WBC) 4.27 × 10^9^/L, red blood cell (RBC) 2.62 × 10^12^/L, hemoglobin (HGB) 79 g/L, platelet (PLT) 24 × 10^9^/L, prothrombin time (PT) 16.20s, activated partial thromboplastin time (APTT) 46.90s, D-dimer >20 mg/L FEU (Table 1). Radiographic data: MRI (magnetic resonance imaging): T11-L1 epidural irregular abnormal signals in the spinal canal, with a range of about 16 mm × 17 mm × 65 mm (Figures 2A–E). Based on the above clinical and imaging presentations, the diagnoses of Thoracolumbar spinal epidural hematoma with bilateral lower limb incomplete paraplegia (ASIA-C, T10-L1), DIC (ISTH overt DIC score: 5 points), Febrile fever of unknown origin (FUO), and Traumatic iliopsoas hematoma formation were made. Hematonosis, infection, autoimmune, syndrome of cauda equina (SCE), or malignancy, such as primary or metastatic epidural tumor, were included in the differential diagnosis. At the time of examination (3 days after onset), reflexes were absent, possibly due to acute cord compression; however, as evolution over time was not documented, further histochemical and histopathological examinations were necessary for differential diagnosis. Given that the patient did not exhibit the typical signs of cauda equina syndrome—which is characterized by a flaccid bladder and overflow incontinence—this diagnosis was excluded. The evacuation of T10-L1 epidural hematoma and expanded decompression of spinal canal were performed. The surgery procedure involved resection of the entire lamina at T10 and the right lamina at T11-L1, revealing, as anticipated from the pre-operative MRI, a hematoma situated on the right side of the spinal canal, which substantially compressed the spinal cord, resulting in minimal dural pulsation (Figure 1B). Then, the evacuated hematoma was sent for routine pathology (Figure 1D). Post-operatively, there was no significant improvement in systemic symptoms. The post-op bone marrow aspiration biopsy showed active proliferation of bone marrow, with more basophils and phagocytes. Unknown cells accounted for 77.00%, and myeloid sarcoma or mast cell leukemia was suspected, while neuroblastoma cell nature could not be excluded. There was a positive immunohistochemical reaction of CD117 and Ki-67, with negative results for the other markers tested in the pathological tissue from the post-op epidural hematoma and bone marrow aspiration biopsy. Combined with flow cytometry, histomorphology, and immunohistochemistry, leukemia was highly suspected. However, as the patient’s condition showed no significant improvement, it was deemed necessary to thoroughly reassess the medical history and conduct a detailed physical examination. It was noted that the child had a large cauliflower-like black mole on the right scalp since childhood, which repeatedly ulcerated and failed to heal (Figure 1A). Subsequently, immunohistochemical staining for MM- related markers on bone marrow and hematoma specimens was conducted, and the results showed positive staining for HMB45, Melan-A, SOX10, S100, and PRAME, with negative results for the other markers. Based on the patient’s scalp lesion, combined with positive immunohistochemical (IHC) staining for tumor markers in both the intraoperative hematoma specimen and the post-op iliac bone marrow biopsy, the results confirmed the diagnosis of MM. However, despite 2 months of aggressive symptomatic and supportive treatment, the child ultimately succumbed to MM forever.

**Table 1:** Emergency laboratory data.

Date: 2024/10/23				
Lab test items	Index	Result	Unit	Reference range
Complete blood count (CBC)	White blood cell count, WBC	4.27	E+9/L	4.1–11.0
	Red blood cell count, RBC	2.62	E+12/L	4.1–5.3
	Hemoglobin, Hb	79	g/L	114–154
	Plateletcrit, PCT	0.03	%	0.11–0.31
Coagulation function	Prothrombin Time, PT	16.2	s	11.00–15.00
	Plasma prothrombin time ratio, PT-R	1.22		0.80–1.15
	International normalized ratio, INR	1.3		0.80–1.20
	Prothrombin time activity, PTA	65	%	80.00–135.00
	Activated partial thromboplastin time, APTT	46.9	s	28.00–43.00
	Fibrinogen, FIB	1.5	g/L	2.00–4.00
	Thrombin time, TT	22.1	s	14.00–21.00
	Factor VIII activity, FVIII:C	174	%	59.00–200.00
	Factor IX activity, FIX:C	114	%	64.00–216.00
Disseminated intravascular coagulation (DIC) panel	D-dimer, D-D	>20	mg/L FEU	0–0.50
	Plasma protamine sulfate-coagulant test	Positive		Negative
	Fibrinogen/Fragment D products, FDPs	>150	mg/L	0–5.00
Liver, kidney function & electrolytes	Alanine aminotransferase, ALT	20	U/L	0–35
	Lactate dehydrogenase, LDH	1,532	U/L	120–246
	Albumin, Alb	44.3	g/L	35–50
	Total bilirubin, TB	32.9	umol/L	8.5–29.2
	Urea	4.79	mmol/L	2.5–7.1
	Creatinine, Cr	37	umol/L	46–92
	Protein in urine, pro	+ −		Negative
	Urobilinogen, UBG	3 +		Negative/Weak positive
	Blood in urine, BLD	3 +		Negative
	Potassium, K^+^	3.92	mmol/L	3.5–5.1
	Sodium, Na^+^	136	mmol/L	137–145
	Calcium, Ca^2+^	2.4	mmol/L	2.10–2.55
	Ferritin, FER	1017.7	ng/ml	4.63–204.0
Infection markers	Interleukin-6, IL-6	32.3	pg/mL	<7.0
	Procalcitonin, PCT	0.72	ng/ml	<0.05
	Erythrocyte sedimentation rate, ESR	40	mm/h	0–20
	High-sensitivity C-reactive protein, hs-CRP	13.34	mg/L	0–10
Thrombosis panel	Thrombin-antithrombin complex, TAT	>120.0	ng/ml	<4.00
	Plasmin-*α*2-antiplasmin-complex, PAP	21.49	ug/ml	<0.80
	Tissue plasminogen activator inhibitor complex, t-PAIC	5.8	ng/ml	3.70–9.30
	Thrombomodulin, TM	8.4	TU/ml	3.80–13.30
Lipid profile	Triglycerides, TG	2.32	mmol/L	0–1.70
	Total cholesterol, TC	6.43	mmol/L	0–5.72
	High-density lipoprotein cholesterol, HDL-C	0.9	mmol/L	0.91–1.55
	Low-density lipoprotein cholesterol, LDL-C	4.33	mmol/L	0–3.64
Thromboelastography (TEG)	Reaction time, R	8.3	min	5–10
	Kinetics, K	3.8	min	1–3
	Alpha angle, α	46.5	deg.	53–72
	Maximum amplitude, MA	45.9	mm	50–70
	Clot strength, G/(d/sc)	4237.9	d/sc	4,500–11,000
	Fecal occult blood test, FOBT	Weak		Negative
		Positive		
	Gastric occult blood test, GOBT	Positive		Negative
Tumor marker panel	Alpha-fetoprotein, AFP	3.4	ng/ml	0–13.4
	Carcinoembryonic antigen, CEA	2.8	ng/ml	0–5.0
	Carbohydrate antigen 19–9, CA19-9	<2.00	U/mL	43.0
	Squamous cell carcinoma antigen, SCC-Ag	0.3	ng/ml	0–3.2

## Discussion

This study presents a rare pediatric case of malignant melanoma (MM) bone marrow infiltration, accompanied by spinal cord compression leading to paraplegia. To date, no previously reported cases have documented pediatric paraplegia secondary to MM bone marrow infiltration. Following the PRISMA guidelines for systematic reviews, a comprehensive and systematic literature review was conducted until March 28, 2025, across multiple databases—including ClinicalKey, Embase, Google Scholar, Medline, Ovid, PubMed, Scopus, and Web of Science—using the search terms “malignant melanoma bone marrow infiltration” OR “malignant melanoma bone marrow metastasis” across all fields. A total of 336,935 publications were identified. After removing duplicate records, 336,718 articles remained. Screening for the publication type “Case Report” filtered the results to 36 articles. Following a full-text review, 3 articles were excluded due to irrelevance to melanoma metastasis. Consequently, 33 studies were included for final analysis (Supplementary Figure 2). A systematic literature review and analysis of 36 cases of MM bone marrow infiltration revealed the following: male 63.89% (23/36) over female 36.11% (13/36). The primary sites of melanoma were distributed as follows: Skin (52.78%, 19/36), Ocular (13.89%, 5/36), Nasal (8.33%, 3/36), Occult (16.67%, 6/36), Retroperitoneal (2.78%, 1/36), Rectal and Vaginal mucosa (2.78%, 1/36), and Not reported (2.78%, 1/36). Hematologic abnormalities were common among these cases. Disseminated intravascular coagulation (DIC) was observed in 11.11% (4/36) of patients. Additional hematologic complications included anemia (19.44%, 7/36), leukocytosis (11.11%, 4/36), thrombocytopenia (27.78%, 10/36), pancytopenia (22.22%, 8/36), coagulopathy (2.78%, 1/36), carcinocythemia (2.78%, 1/36), microangiopathic hemolytic anemia (2.78%, 1/36), leucoerythroblastic picture (8.33%, 3/36), and hypercalcemia (2.78%, 1/36). The prognosis involvement remains extremely poor, with 58.33% (21/36) of patients succumbing to the disease within days to months following diagnosis. Notably, only three patients demonstrated favorable therapeutic responses, with two patients achieving complete remission or significant clinical improvement following nivolumab/ipilimumab therapy and one patient responding positively to pembrolizumab. Additionally, the overall prognosis is extremely poor, with the majority of patients (58.33%, 21/36) succumbing to the disease within days to months following diagnosis, despite undergoing chemotherapy or supportive treatment. Notably, two patients achieved complete relief after treatment with Nivolumab/Ipilimumab, while one patient exhibited good response to Pembrolizumab (Table 2). Unfortunately, this patient did not receive post-discharge immunotherapy with either nivolumab/ipilimumab combination or pembrolizumab monotherapy. In addition, Shimizu et al. (9). conducted a systematic review of 29 studies and record that bone metastatic melanoma accounted for 13% (3,130/23,998) with a male-to-female distribution of 56 to 44%. Further analysis of 21 studies revealed the prevalence of bone pain in 3.48% (180/5167), total skeletal-related events (SREs) in 26.88% (1,389/5167). Among the SREs events, pathological fracture occurred in 6.93% (358/5167), spinal cord compression in 2.88% (149/5167), hypercalcemia in 0.74% (38/5167), radiotherapy in 11.83% (611/5167), and surgery to the bone in 9.72% (502/5167).

**Table 2:** Systematic review and comprehensive analysis of bone marrow infiltration in malignant melanoma.

Authors	Year	Age	Gender	Symptoms	Primary	Treatment	Outcome
Franklin JW (10)	1952	18	M	Fatigue, Lt. pleural effusion	Skin (Lt. angle of the jaw)	NA	Unknown
Franklin JW (10)	1952	67	M	Back pain	Skin (Back)	Supportive	Death
Gallivan MV (11)	1984	55	F	Abdominal pain, carcinocythemia	Retroperitoneal	Chemotherapy	Death, months
Brown D (12)	1990	66	M	Malaise, weight loss	Ocular	Supportive	Death, months
Basile M (13)	1992	63	M	Thrombocytopenia	Skin lesion? (Lt. axillary mass)	Chemotherapy	Death, 6 months
Villarrubia J (14)	1995	57	F	Petechiae, leukoerythroblastic reaction, thrombopenia	Skin (Lt. knee)	NA	Unknown
Bhagwati N (15)	1998	34	M	Microangiopathic hemolytic anemia, DIC	Occult	Supportive	Death, 3 weeks
Trefzer U (16)	1999	48	M	Fatigue, weight loss, dyspnea, limb pain; Thrombocytopenia, anemia, leucocytosis	Occult	NA	Death, 9 days
Invernizzi R (17)	2001	34	M	Fever, hepatosplenomegaly; Thrombocytopenia, anemia	Skin (Rt. ear)	NA	Unknown
Chim CS (18)	2001	67	M	Painful right eye with blurring of vision, hepatosplenomegaly; Leucoerythroblastic picture	Ocular	NA	Unknown
de Wolff JF (19)	2004	63	F	Fatigue, dyspnea; Leucocytosis, splenomegaly	Skin (Lt. ankle)	Supportive	Death, days
Spiller SE (4)	2005	3	M	Pancytopenia	Skin	Chemotherapy	Death
Uesawa M (20)	2006	67	F	Back pain	Skin (Rt. maxillary bone)	NA	Unknown
Wong KF (21)	2006	74	F	Pancytopenia	Rt. nasal cavity	NA	Unknown
Batsis JA (22)	2006	75	F	Progressive right-sided weakness, vomiting, constipation, decreased appetite and central abdominal discomfort; Leucocytosis, hypercalcaemia	Skin (neck)	Supportive	Death, 3 weeks
Jain D (23)	2007	22	M	Epistaxis; Anemia, thrombocytopenia	Occult	Chemotherapy	Unknown
Bhandari S (24)	2009	62	M	Generalized aches and pains; Leucoerythroblastic, anemia, thrombocytopenia	Ocular	Supportive	Unknown
Bertolotti A (25)	2012	52	F	Dyspnea; anemia, thrombocytopenia	Skin	Supportive	Death, days
Serrier C (26)	2013	60	M	Dyspnea, back pain	Skin	Supportive	Death, days
Velasco-Rodríguez D (27)	2013	75	M	Epistaxis; Pancytopenia	Skin	Chemotherapy	Death, 2 months
Hsiao SY (28)	2014	76	M	Back pain	Ocular	Supportive	Death, 2 months
Suzuki T (29)	2014	77	M	Thrombocytopenia, leukocytosis, DIC	Ocular	Supportive	Death, 1 week
Rosner S (30)	2015	64	M	Fatigue, fever; Pancytopenia	Skin	Pembrolizumab	Good response
Volejnikova J (31)	2016	5	M	Back and leg pain, fever	Skin	Ipilimumab	Death, 4 months
Kassam S (32)	2016	79	F	Weight loss	Occult	Supportive	Death, 2 weeks
Fukumoto T (33)	2016	29	M	Back pain	Skin	Nivolumab	Death, 3 months
Gbadamosi B (5)	2018	30	F	Fatigue, back pain, fever; Thrombocytopenia, anemia, coagulopathy, DIC	Skin (Scalp)	Nivolumab/Ipilimumab	Complete relief
Bain BJ (34)	2019	33	M	Rt. axillary lymphadenopathy	Skin (Rt. Shoulder)	NA	Unknown
John VM (35)	2019	69	F	A small, painless black swelling on the dorsal aspect of Lt. foot; Pancytopenia	Skin (Lt. foot)	Supportive	Death, 1 week
Ruchee Khanna (36)	2020	50	M	Fatigue, back pain; Anemia, thrombocytopenia, leucoerythroblastic	Rectal and vaginal mucosa?	NA	Unknown
Luna Pais H (37)	2021	79	F	DIC	Lt. nasal	Nivolumab/Ipilimumab	Complete relief
Jain M (38)	2022	40	M	Abdominal distention, fever, ill-defined swelling over the sternum	Occult	NA	Unknown
Frioni F (39)	2022	56	F	Epistaxis, petechiae	Nasal	NA	Unknown
Rokkam VRP (40)	2023	35	F	Pancytopenia	Not reported	Supportive	Death, 16.5 weeks
Rokkam VRP (40)	2023	66	M	Pancytopenia	Skin (Back)	Supportive	Death, 13.5 weeks
Rokkam VRP (40)	2023	67	M	Rt. hip pain; Persistent cytopenia	Occult	Supportive	Death, 9.5 weeks

DIC, disseminated intravascular coagulation; F, female; Lt., left; M, male; NA, not available; Rt., right.

Melanoma is the third most prevalent skin cancer globally, accounting for 1–3% of all malignant tumors. In recent years, the incidence has been steadily rising, with a higher prevalence in males than females. It is projected that by 2040, the number of cases will reach 510,000, with an estimated 96,000 deaths (41–43). Among pediatric melanoma patients, the majority are diagnosed post-puberty (44). MM arised from neuroectoderm-derived pigment-producing cell, which is initiated and driven to migrate to other sites by environmental, genetic, constitutional, and epigenetic factors, as well as acquired mutations, during embryonic development, involves skin (13–38%), distant lymph nodes (5–34%), lung (18–36%), liver (14–20%), central nervous system (2–20%), bones (4–17%), gastrointestinal tract (1–8%), adrenal glands (1–11%), pancreas (3%), pleura (3%), heart (<1%), kidneys (<1%), and thyroid (<1%) (45, 46).

There are four major clinical-pathological subtypes of melanoma: superficial spreading melanoma (SSM), nodular melanoma (NM), acral lentiginous melanoma (ALM), and lentigo malignan melanoma (LMM). In early stages, melanoma exhibits a highly metastatic potential, capable of spreading via lymphatic, hematogenous (3, 47). In the head and neck, it can manifest as recurrent epistaxis, anosmia, and headache. Spinal cord metastasis typically manifests as back pain and symptoms of spinal cord compression. Bone marrow infiltration may present with symptoms such as pain, anemia, thrombocytopenia, bone marrow failure, leukoerythroblastosis, pancytopenia, and leukopenia, with DIC in 11% of cases (4, 48). In the case we reported, the children had a pigmented mass on the right scalp since childhood. However, due to inadequate attention, the lesion repeatedly ulcerated. The children later experienced spontaneous epistaxis accompanied by extensive ecchymosis after physical exercise. Multiple blood tests consistently revealed DIC, and both bone marrow aspiration and intra-operative hematoma pathological biopsy showed that it was consistent with the diagnosis of MM bone marrow infiltration. Regrettably, as documented in the literature, the prognosis of bone marrow metastasis is extremely poor, with a median survival time of only 2 months (48).

To our knowledge, this case is exceptionally rare, as DIC was triggered by MM bone marrow infiltration, and even more unusual is the presence of SEH within the thoracolumbar spinal canal on MRI. SEH is a rare yet potentially fatal disease, with an incidence <1/1000000, accounting for about 1% of spinal epidural space-occupying lesions (49). Over the past 3 years, around 1,000 cases have been documented in the literature worldwide. Common etiologies include trauma, coagulopathy, drugs, hypertension, arteriovenous malformations or spinal puncture. Hematoma typically occur in the dorsal cervicothoracic and thoracolumbar segments, with typically manifests as patients severe knife pain at the site of hemorrhage, and some may experience painless intervals, followed by progressive paralysis below the affected spinal cord level (7, 8, 50). MRI is the preferred imaging modality when SEH is suspected. In acute phase (<24 h), T1-weighted images showed isointensity, while T2-weighted images demonstrate hyperintensity. In subacute phase (>24 h), both T1 and T2-weighted images showed hyperintensity. In chronic phase, both T1 and T2-weighted images showed hypointensity. Fat-saturation proton density-weighted images were used to distinguish hematoma from epidural fat (51). A consensus from most reported cases suggests that once SEH was diagnosed, emergent or at least urgent surgical intervention is necessary, with earlier intervention leading to a better prognosis. Besides, pre-operative neurologic status is also a critically important prognostic indicator (50, 52–54). Therefore, once MM bone marrow infiltration with SEH was diagnosed, surgery (hemilaminectomy or laminectomy with irrigation and debridement) or conservative management based on the imaging data and neurologic status should be selected as soon as possible (52). In this case, despite urgent surgical intervention and subsequent systemic treatment for MM, the outcome was unfortunately unsatisfactory, and succumbed to the disease, after 2 months.

## Conclusion

In conclusion, MM bone marrow infiltration induced coagulation dysfunction and SEH with paraplegia is a rare and complex clinical disease, that can be highly challenging to diagnose by pre-operative image. Therefore, we recommend repeated systematic and comprehensive medical history and physical examination, especially neurologic assessment, which is necessary, and urgent MRI is essential for accurate differential diagnosis, and multidisciplinary consultations should be considered when necessary. Once diagnosed, prompt surgical intervention is critical, as early intervention is associated with a significantly favorable prognosis. Besides, given the aggressive nature of MM bone marrow infiltration, early recognition and prompt intervention are paramount. Further research is warranted to better understand the pathophysiology, optimize therapeutic strategies, and improve patient outcomes.

## Data Availability

The original contributions presented in the study are included in the article/Supplementary material, further inquiries can be directed to the corresponding author.

## Supplementary material

SUPPLEMENTARY FIGURE 1 Diagnosis and treatment schedule.

SUPPLEMENTARY FIGURE 2 Literature search and screening flow chart (PRISMA).
