# Supplementary figures and images for: Malignant melanoma bone marrow infiltration induced coagulation dysfunction and spinal epidural haematoma with paraplegia: a case report and literature review
Source: Front Med (Lausanne). 2025 Oct 27;12:1601774. doi: 10.3389/fmed.2025.1601774 (PMC12597949; doi:10.3389/fmed.2025.1601774)

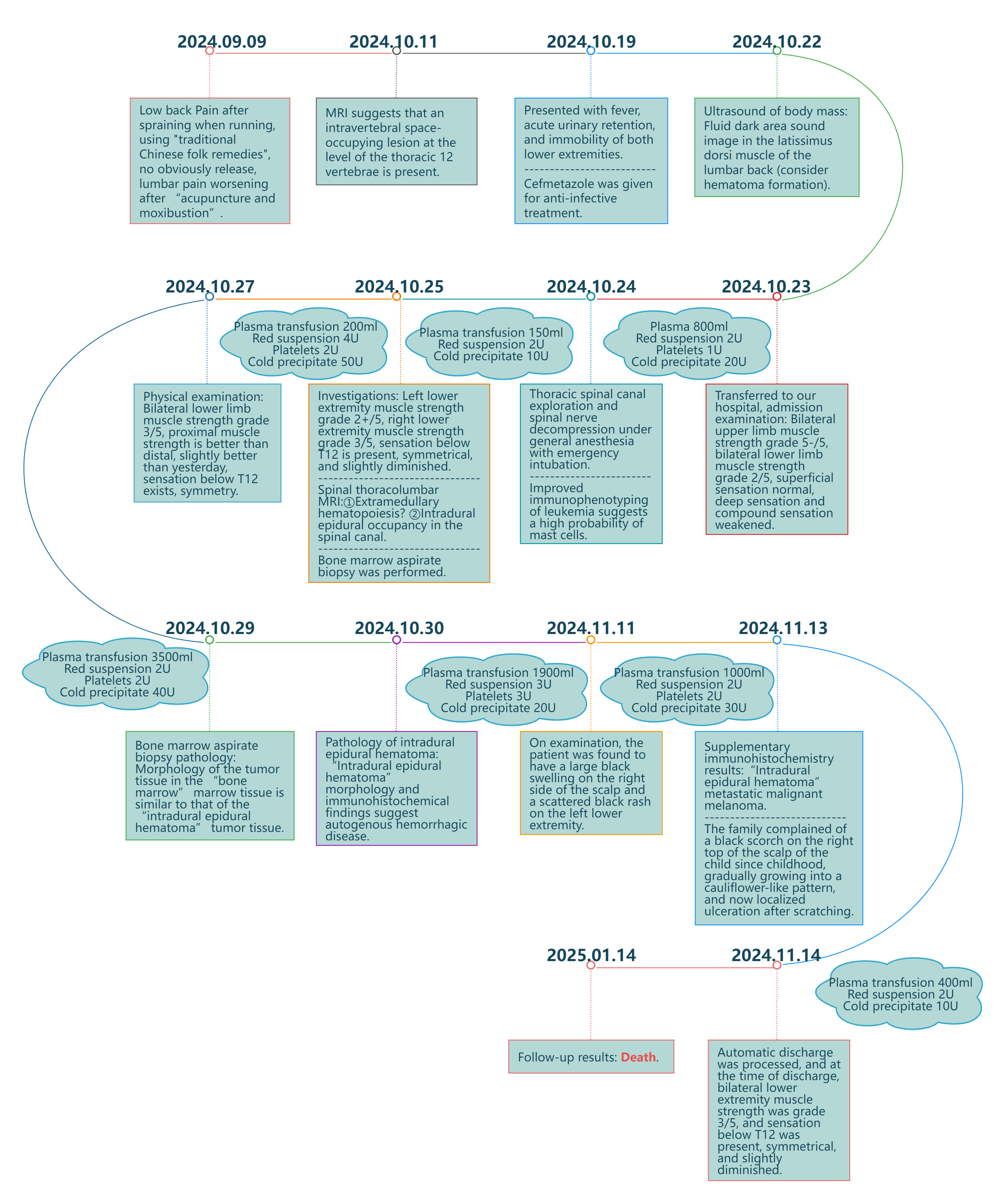

Supplement: SUPPLEMENTARY FIGURE 1 — Diagnosis and treatment schedule. [file Image_1.png]

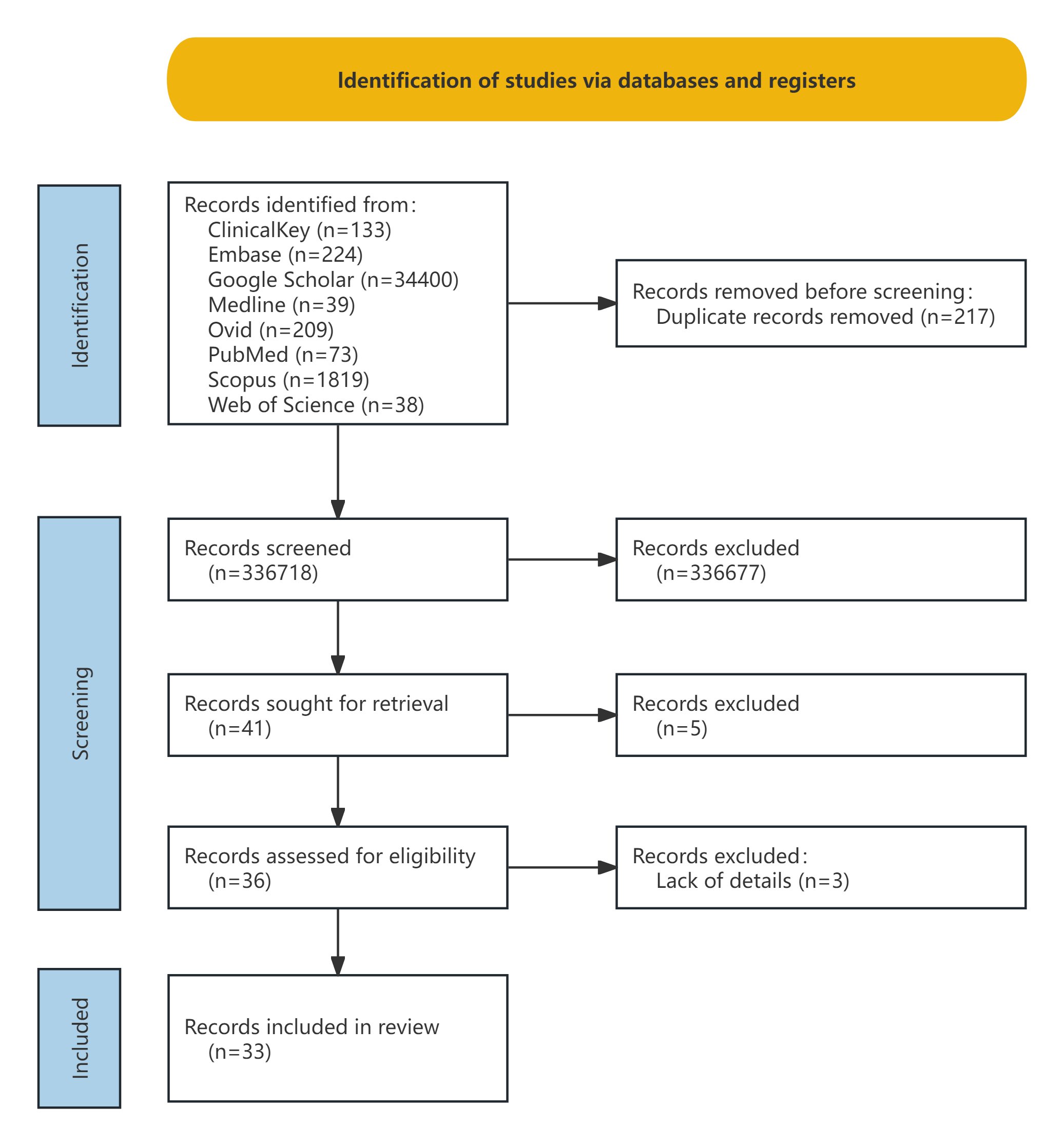

Supplement: SUPPLEMENTARY FIGURE 2 — Literature search and screening flow chart (PRISMA). [file Image_2.png]
